# Toll-like receptor 4 agonist and antagonist lipopolysaccharides modify innate immune response in rat brain circumventricular organs

**DOI:** 10.1186/s12974-019-1690-2

**Published:** 2020-01-06

**Authors:** Alejandra Vargas-Caraveo, Aline Sayd, Javier Robledo-Montaña, Javier R. Caso, José L. M. Madrigal, Borja García-Bueno, Juan C. Leza

**Affiliations:** 1 Departamento de Farmacología y Toxicología, Facultad de Medicina, Universidad Complutense de Madrid (UCM); Centro de Investigación Biomédica en Red de Salud Mental (CIBERSAM); Instituto de Investigación Sanitaria Hospital 12 de Octubre (Imas12); Instituto Universitario de Investigación en Neuroquímica UCM, Avda. Complutense s/n, 28040 Madrid, Spain; 2 Biological and Health Sciences Division, Metropolitan Autonomous University (UAM), Campus Lerma, 52005 Lerma, Mexico

**Keywords:** Innate immunity, Blood-brain interfaces, Bacterial infection, Microglia, Immunofluorescence

## Abstract

**Background:**

The circumventricular organs (CVOs) are blood-brain-barrier missing structures whose activation through lipopolysaccharide (LPS) is a starting point for TLR-driven (Toll-like receptors) neuroinflammation. The aim of this study was to evaluate in the CVO area postrema (AP), subfornical organ (SFO), and median eminence (ME), the inflammatory response to two TLR4 agonists: LPS from *Escherichia coli* (EC-LPS), the strongest endotoxin molecule described, and LPS from *Porphyromonas gingivalis* (PG-LPS), a pathogenic bacteria present in the periodontium related to neuroinflammation in neurodegenerative/psychiatric diseases. The response to LPS from the cyanobacteria *Rhodobacter sphaeroides* (RS-LPS), a TLR4 antagonist with an interesting anti-inflammatory potential, was also assessed.

**Methods:**

LPSs were intraperitoneally administered to Wistar rats and, as indicatives of neuroinflammation in CVOs, the cellular localization of the nuclear factor NF-κB was studied by immunofluorescence, and microglia morphology was quantified by fractal and skeleton analysis.

**Results:**

Data showed that EC-LPS increased NF-κB nuclear translocation in the three CVOs studied and PG-LPS only induced NF-κB nuclear translocation in the ME. RS-LPS showed no difference in NF-κB nuclear translocation compared to control. Microglia in the three CVOs showed an ameboid-shape after EC-LPS exposure, whereas PG-LPS only elicited a mild tendency to induce an ameboid shape. On the other hand, RS-LPS produced a markedly elongated morphology described as “rod” microglia in the three CVOs.

**Conclusions:**

In conclusion, at the doses tested, EC-LPS induces a stronger neuroinflammatory response than PG-LPS in CVOs, which might be related to their different potency as TLR4 agonists. The non-reduction of basal NF-κB activation and induction of rod microglia by RS-LPS, a cell morphology only present in severe brain injury and infections, suggests that this molecule must be carefully studied before being proposed as an anti-inflammatory treatment for neuroinflammation related to neurodegenerative/psychiatric diseases.

## Background

Circumventricular organs (CVOs) are brain structures of the third and fourth ventricles. They are characterized by the absence of blood-brain-barrier (BBB) and are highly vascularized with fenestrations. The area postrema (AP) is present in the fourth ventricle and the subfornical organ (SFO) in the third ventricle, both of which are considered sensory CVOs by the presence of neuronal soma and terminals that sense blood and intestinal fluids and have connectivity to other brain areas with BBB [1]. Median eminence (ME) is considered a secretory CVO located in the basal hypothalamus with a fenestrated endothelium [2].

Because of the lack of BBB, CVOs are exposed to circulating LPS [3]. It has been established that these structures have basal expression levels of LPS receptors CD14 and TLR4 [4]. Recently, our group has demonstrated that lipid A and core oligosaccharide, key regions of the LPS molecule, are present in physiological conditions in AP, SFO, and ME, in constant co-localization with CD14 and TLR4 [5]. Although several studies have used intraperitoneal and cerebroventricular injections of *E. coli* LPS as a model of neuroinflammation [4, 6, 7], it is important to point out that, in stress-related neuropsychiatric disorders, LPS can be detected in the bloodstream in higher concentrations than in healthy controls [8, 9]

LPS toxicity depends on lipid A composition, in particular, the acyl chain region of the lipid A moiety from LPS is recognized by CD14 and TLR4 receptors in most cells, triggering the innate immune signaling pathway, inducing NF-κB nuclear translocation and, consequently, the release of pro-inflammatory cytokines and the synthesis of inducible inflammatory and oxido/nitrosative enzymes [5]. Therefore, the lipid A structure is related to its endotoxic properties, but some differences in potency have been described, depending on the microbial species, environmental conditions such as temperature and interactions with the host immune system [10–12]. For example, the lipid A moiety produced by Enterobacteriaceae, and other gram-negative aerobes, has 6 fatty acyl chains and 2 phosphates, and this is excellent for binding to TLR4/MD2 complex and promoting dimerization and activation. The TLR4 signal transduction produced by a hexaacyl lipid A, e.g., from *E. coli* (EC-LPS) like the one used in this study, is characterized by a strong pro-inflammatory signal leading to a high expression of tumor necrosis factor α (TNF-α), interleukin-1β (IL-1β), macrophage inflammatory protein 2 (MIP-2), interleukin 12 p40 (IL-12 p40) and interferon γ (IFN-γ) [13].

Lipid A structures synthesized by other families of bacteria, with less fatty acyl chains and/or lack of 4’-phosphate are considered weak TLR4 agonists or TLR4 antagonists [12, 14]. An excellent example of this is the LPS from *Porphyromonas gingivalis* (PG-LPS), a weak TLR4 agonist, with a pentaacyl lipid A, less endotoxic properties compared to EC-LPS that induces the expression of TNF-α, IL-1β, and MIP-2, but not IL-12 p40 and IFN-γ [13], but with significant relevance in the inflammatory response in periodontal disease. Recently, inflammation derived from periodontitis has been related to the neuroinflammation state in neurodegenerative [15] and psychiatric diseases [16, 17]. Oral infection with *P. gingivalis* in mice can produce an impairment of learning and memory abilities by the release of pro-inflammatory cytokines in the brain [18], as well as depression-like behavior and a reduction of brain-derived neurotrophic factor (BDNF )[19]. A PG-LPS i.p. injection in rodents can lead to impairments in learning and memory tasks, and to an increase of inflammatory cytokines (TNF-α, IL-1β, IL-6, and IL-8) in brain cortex and activation of microglia and astrocytes in both hippocampus and brain cortex [20].

On the other hand, a potent TLR4 antagonist in rodents and humans is the LPS synthetized by the cyanobacteria *Rhodobacter sphaeroides* [21]. Lipid A of RS-LPS does not induce cytokine expression because its signal transduction is not carried out; consequently, NF-κB is not translocated to the nucleus [13]. This bacterium has no relevance as a pathological microorganism; however, its LPS has been widely used as a blocker of TLR-4 to prevent inflammatory response in the presence of the highly endotoxic EC-LPS and it has been proposed as a neuroprotective strategy to prevent neuroinflammation [22, 23]. Microglial activation by EC-LPS in slices of the corpus callosum was correlated with axonal malfunction and with the accumulation of β-amyloid precursor protein in nerve fibers and a double treatment with EC-LPS and RS-LPS inhibited TLR-4 pathway activation and reversed microglial activation [24]. Using RS-LPS in an experimental model of epilepsy to block TLR-4 and to inhibit the release of pro-inflammatory mediators by microglia and astrocytes, excitability decreased in seizure threshold [25]. In experiments of nociception through chronic constriction injury to the sciatic nerve, the blockade of TLR-4 using RS-LPS by repeated intrathecal administration, attenuated allodynia and hyperalgesia [26]. On the contrary, in mouse primary microglia cultures, it was observed that RS-LPS can develop TLR-4 agonistic properties inducing the release of cytokines and chemokines [27].

Based on this evidence*,* we suggest that the presence of LPS molecules in the bloodstream derived from different gram-negative species and with distinct endotoxic properties modifies immediately the activity of the innate immune-mediated intracellular pathways in the first line of immune defense in the brain, the CVO’s. The present study aimed to evaluate the effects on CVOs neuroinflammatory response (NF-κB and microglia activation) of the weak TLR4 agonist PG-LPS and the TLR4 antagonist RS-LPS in comparison with the very well-known neuroinflammatory trigger and strongest TLR4 agonist EC-LPS.

## Methods

### Animals

Sixteen male Wistar Hanover rats (HsdRccHan:Wist, from Envigo, Spain), weighing 250-280 g were used. The rats were housed individually with standard temperature and humidity conditions and in a 12-h light/dark cycle (lights on at 8:00 h) with free access to food and water. All the animals were maintained under these conditions for 7 days prior to the experiment.

### Experimental groups and LPS administration

Four experimental groups were used for the treatment with three different types of LPS and a control group (vehicle-injected). EC-LPS (*Escherichia coli* serotype 0111: B4, ref. L2630, Sigma-Aldrich, Spain), PG-LPS (*Porphyromonas gingivalis* ref. tlrl-pglps, InvivoGen USA) and RS-LPS (*Rhodobacter sphaeroides* ref. tlrl-rslps, InvivoGen, USA) were dissolved in sterile 0.9% saline solution. Group sizes were previously calculated using specialized software (STATGRAPHICS Centurion XVI, UCM license). The number of animals (*n* = 4) is the one obtained when, based on previous data from our laboratory and other groups performing immunohistochemistry analysis on counting cells in CVO [28], the difference to detect is set at 3 times (3×) the sigma, with a test potency of 80% and an alpha risk of 0.05.

The rats were intraperitoneally injected with 0.5 mg/kg of LPS or 2 mL/kg of saline solution, respectively. Ninety minutes later, animals were sacrificed using sodium pentobarbital (320 mg/kg i.p.).

Timing and dose of LPSs i.p. injections were chosen based on previous studies, where a non-endotoxemic dose of EC-LPS at 0.5 mg/kg was used to induce mild neuroinflammation after 90 min [29, 30]. Other relevant studies that evaluate inflammatory response in the CVOs have used EC-LPS doses from 50 μg to 1 mg/kg at different times from 30 min to 4 h [31, 32]. Our model is an acute LPS administration and we decided to use 90 min to observe an immediate effect on the CVOs, the first line of brain immune defense [33]. It has been reported that after 3 h, other structures start to develop the inflammatory response and CVOs stop their response [33].

Since there are only a few works related to the effects of PG-LPS and RS-LPS on the brain, and, to our knowledge, none of them has analyzed the inflammatory response in the CVOs, we decided to extend the same timing and dose that we have previously used for EC-LPS to PG-LPS and RS-LPS in order to compare their effects on innate immune response.

### Immunohistochemistry

Transcardial perfusion was performed through the left ventricle with 200 mL of saline solution, and the right atrium was opened. Next, 200 mL of 4% paraformaldehyde (PFA) in 0.1 M of PBS (pH 7.4) was perfused. Brains were dissected and post-fixed in 4% PFA overnight at 4 °C, then were equilibrated in 30% sucrose at 4 °C until precipitate appeared, around 48 h. Brains were embedded in an optimum cutting temperature (O.C.T. tissue tek) and frozen at −20 °C to obtain 15-μm coronal sections using a cryostat. Consecutive sections were obtained from the SFO (between Bregma −0.84 and −1.08 mm), ME (between Bregma −1.80 and −2.04 mm), and AP (between Bregma −13.68 mm to 14.16 mm) by using as reference the stereotaxic atlas of Paxinos and Watson. Sections were collected and mounted on slides with adherent coating. All brain tissue sections were maintained at −40 °C until use.

Simultaneous double immunostaining was performed in three consecutive sections for each structure. Sections were washed with KPBS 0.02 M for 5 min, treated with 0.1 M of glycine during 20 min to eliminate autofluorescence, two 5-min washes with KPBS, then the blocking process for 30 min with 10% BSA in KPBS. Incubation with primary antibodies, in KPBS containing 10% BSA, lasted 2 h, at room temperature. Sections were washed in KPBS three times for 5 min each; secondary antibody incubation was carried out for 1 h, antibodies were diluted in KPBS (pH 7.4) containing 10% BSA; thereafter, sections were washed three times in PBS for 5 min each, and one last wash with deionized water alone for 5 min. Finally, 4′,6-diamidino-2-phenylindole dihydrochloride (DAPI) containing Fluoroshield (Sigma Aldrich) mounting medium was added to the slides in the double immunostaining procedure. Sections were coverslipped and frozen at −20 °C or immediately visualized using a high-performance fluorescence microscope. Confocal images were obtained in the confocal Olympus microscope FV1200, from CAI-UCM Centro de Citometría y Microscopía de Fluorescencia.

Titration for every primary and secondary antibody was tested at different dilutions to avoid nonspecific interactions but establishing the best immunosignal. In order to corroborate if nonspecific binding of the fluorescent secondary antibodies was present, negative controls were carried out, for each antibody, using the sample protocol, instead of primary antibody incubation, tissue was maintained with blocking solution during this time.

### Image analyses

Quantification of cells with nuclear translocation and retention of NF-κB and number of total cells of the section with DAPI staining in AP and SFO structures were performed using the 3D objects counter tool for automated counting Fiji. For ME structure, quantification was made manually using the cell counter plugin software because only the area with fenestrated vessels was considered. Three consecutive sections of each CVO, from each rat, were analyzed. Images were processed to adjust brightness, contrast and merge images in the processing package “Fiji”.

In each section, overlapping of red and blue fluorescent signals was considered as nuclear translocation and a red immunosignal surrounding DAPI staining was considered as NF-κB cytoplasmic retention, using a cell counter plugin as well. The percentage of cells with NF-κB nuclear translocation or NF-κB cytoplasmic retention was determined normalizing the total number of cells with NF-κB nuclear translocation by the number of cells in the control group.

A microglia morphology analysis was performed based on [34]. Fractal and skeleton analyses were carried out from binary (black and white) images. Using a Fiji image J® package, several steps were followed to apply commands and options in order to obtain binary images of microglial cells from microphotographs obtained using the epifluorescence microscope. Immunofluorescence with an Iba-1 marker was used to obtain a ×40 microphotograph in grayscale. At least 5 cells from each organ, from each rat, were evaluated with this protocol. Those cells with a complete DAPI staining signal were only included for the analysis. Brightness and contrast were adjusted and an unsharp mask option was applied. To remove “salt and pepper noise”, the despeckle filter was applied. To convert the image into binary format, the threshold option was used and adjusted as needed and the noise was subsequently eliminated using despeckle and remove outliers. Then, each cell was selected to be analyzed with the rectangle tool using the region of interest (ROI) to apply the same size rectangle for all selected cells and duplicated in a new window. With the brush tool, the extra signal was removed to leave a single-cell image and later saved as a new file. The new single-cell binary file was used to convert it into an outline or skeletonized format to carry out a fractal and skeleton analysis, respectively.

A fractal analysis was carried out using the plugin FracLac sitting Num G to 4 and the metrics box on the outlined cell from the binary image was checked. Then, the scan was run to obtain the hull and circle results selecting data of interest such as span ratio, radius, circularity and perimeter, and lacunarity was selected from the Box count summary. These parameters allowed us to determine shape differences between LPS treatments to indicate whether a cell was elongated or round. Skeleton analysis was performed on a cell-skeletonized image. The plugin skeleton-analyze skeleton was selected, and the branch information box was checked. The results obtained show information on the number of branches, junctions, and endpoint voxels.

### Statistical analyses and antibodies

Data are expressed as means (3 consecutive sections for the NF-kB p65 nuclear translocation analysis or for the number of microglial cells analyzed morphologically) of the means (4 animals) ±. For comparisons, a one-way ANOVA followed by the Tukey post hoc test to compare means between groups was carried out. A *p* value of < 0.05 was considered statistically significant.

The following antibodies were used: NF-κB p65 was detected with the rabbit monoclonal IgG (8242, Cell Signaling, dilution 1:200) and visualized with the Alexa Fluor 555 conjugate Donkey anti-rabbit (A31572, Life Technologies, dilution 1:1000). Iba-1 was detected with the goat polyclonal IgG (ab5076, Abcam, dilution 1:500) and visualized with Alexa fluor 488 conjugated Donkey anti-goat (A11055, Life Technologies, dilution 1:1000). CD163 was detected with the mouse monoclonal IgG (sc-58965, Santa Cruz, dilution 1:300) and CD45 mouse monoclonal IgG1 (sc-53045, Santa Cruz, dilution 1:200) both visualized with the secondary the Alexa fluor 488 conjugate Donkey anti-mouse IgG (A21202, Life Technologies, dilution 1:1000). NeuN was detected and visualized with the Alexa Fluor®488 conjugated mouse monoclonal IgG (MAB377X, Millipore, dilution 1:400). All the primary antibodies were previously proved for their specific antigens [5, 35, 36].

## Results

NF-κB is located in different cellular compartments of CVO cells depending on the TLR4 agonist and antagonist lipopolysaccharides administered.

Immunofluorescence images obtained from AP, SFO, and ME of the different LPS treatments showed that NF-κB was retained in the cytoplasm, surrounding the nucleus or translocated into the nucleus, and these events changed according to each experimental group.

**Fig. 1:**
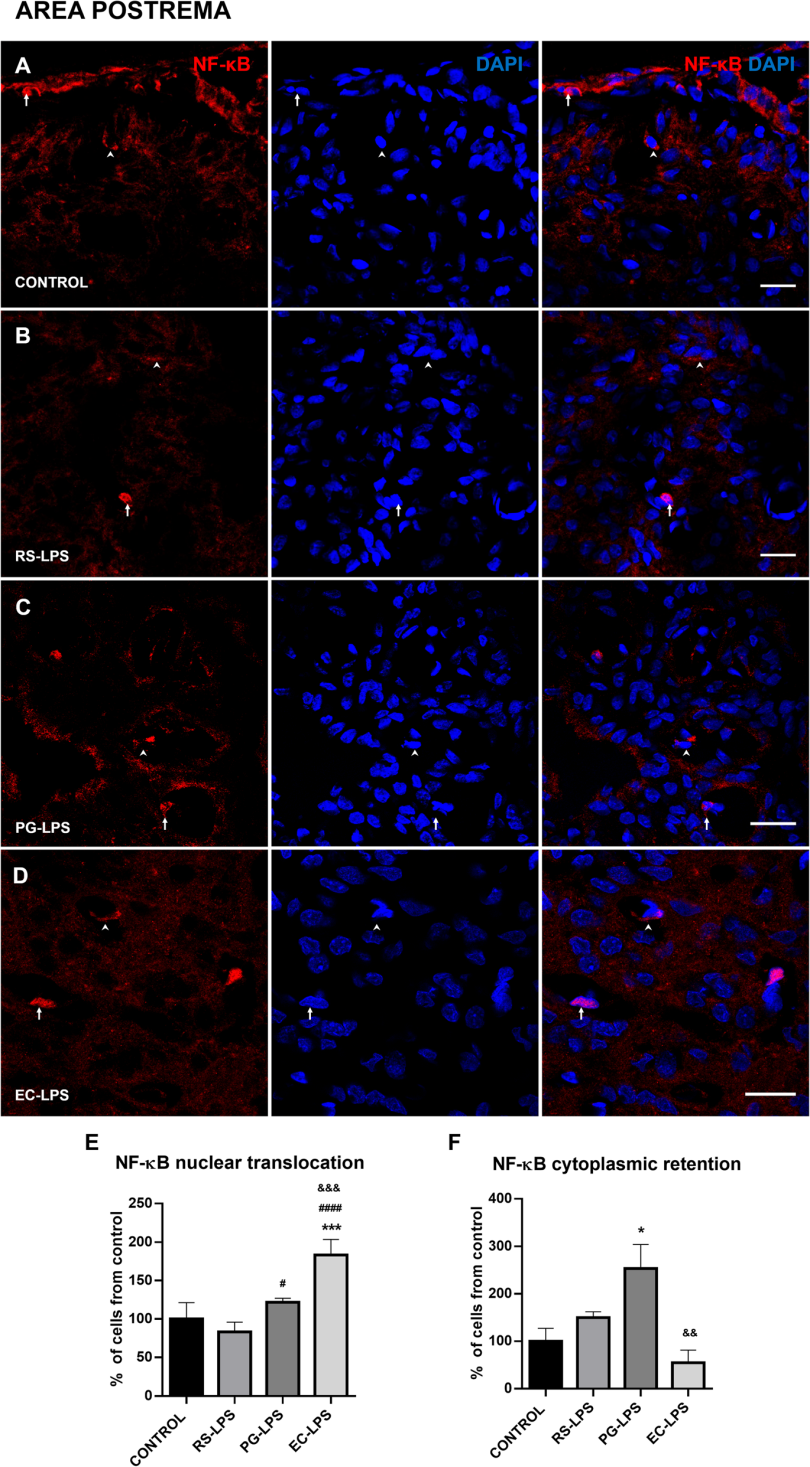
NF-κB cellular localization in the AP of rats i.p. treated with TLR-4 agonists and antagonist LPS. Immunofluorescences of NF-κB in rat AP sections were performed. **a** The control group, **b** RS-LPS i.p. group, **c** PG-LPS i.p. group, and **d** EC-LPS i.p. group. In all cases, red corresponds to NF-κB immunosignal and blue DAPI staining in the nucleus. Arrows indicate red and blue immunosignal overlapping or NF-κB nuclear translocation. Head arrows indicate red immunosignal surrounding blue signal NF-κB cytoplasmic retention. Scale bars = 20 μm. **e** Quantitative analysis of the percentage of NF-κB nuclear translocation. **f** Quantitative analysis of the percentage of NF-κB cytoplasmic retention. Data (*n* = 4) were expressed as the mean (± SEM). *p < 0.05, ***p < 0.001, vs control. #*p* < 0.05, ###*p* < 0.001 vs RS-LPS. &*p* < 0.01, &&&*p* < 0.001 vs PG-LPS. One-way ANOVA with Tukey’s post hoc test

A quantitative analysis revealed that treatment with EC-LPS induced an increment of NF-κB nuclear translocation in the AP compared to control (*p* = 0.0001), PG-LPS (*p* = 0.0008), and RS-LPS (*p* < 0.0001) groups. PG-LPS and RS-LPS treatments tended to increase and decrease the number of cells with NF-κB nuclear translocation compared to EC-LPS, respectively (Fig. 1e).

NF-κB cytoplasmic retention was observed in the three treatments and in the control group. In the AP, retention in PG-LPS was significantly greater than control and EC-LPS groups (Fig. 1f).

**Fig 2:**
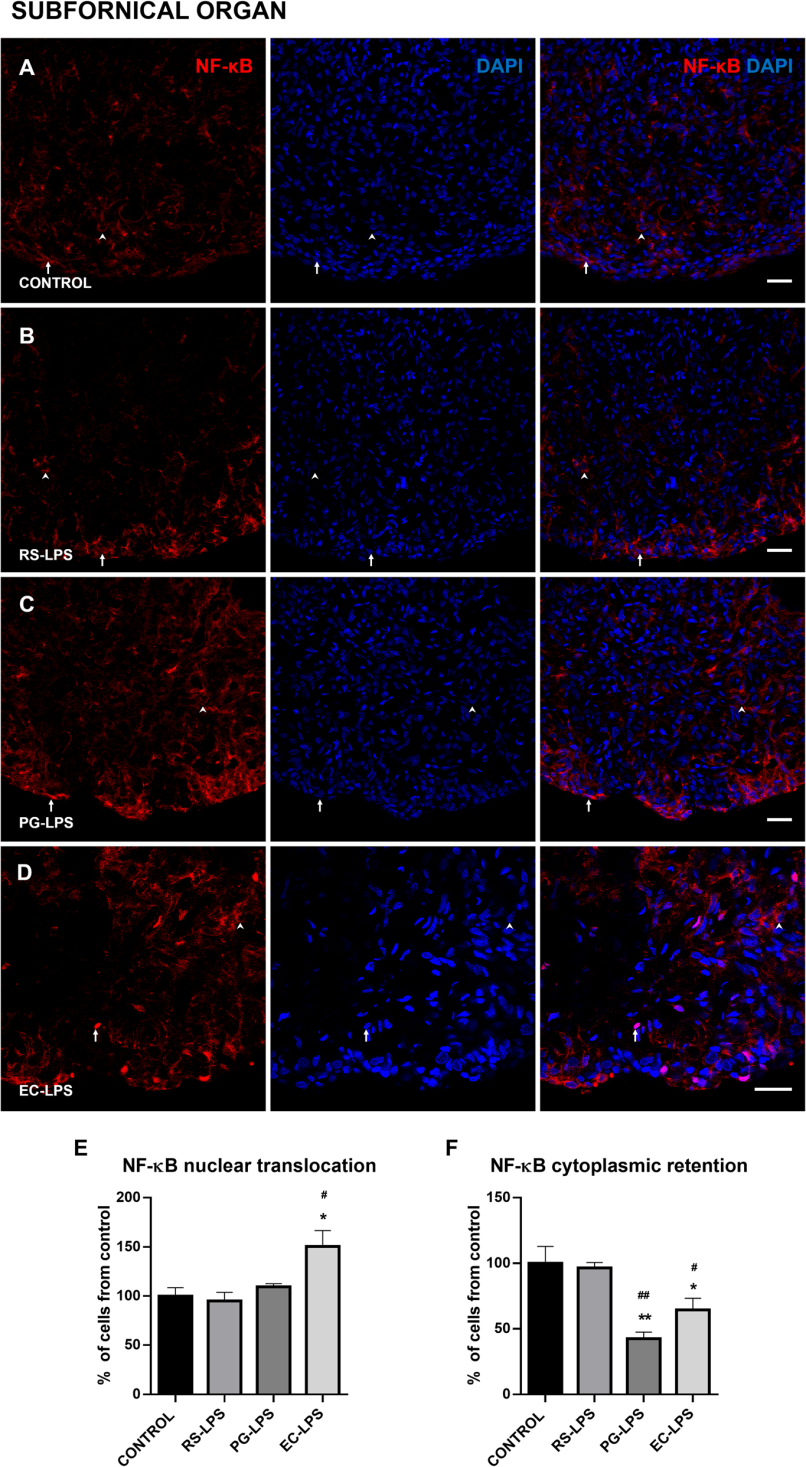
NF-κB cellular localization in the SFO of rats i.p. treated with TLR-4 agonists and antagonist LPS. Immunofluorescences of NF-κB in rat SFO sections were performed. **a** The control group, **b** RS-LPS i.p. group, **c** PG-LPS i.p. group, and **d** EC-LPS i.p. group. In all cases, red corresponds to NF-κB immunosignal and blue DAPI staining in the nucleus. Arrows indicate red and blue immunosignal overlapping or NF-κB nuclear translocation. Head arrows indicate red immunosignal surrounding blue signal NF-κB cytoplasmic retention. Scale bars = 20 μm. **e** Quantitative analysis of the percentage of NF-κB nuclear translocation. **f** Quantitative analysis of the percentage of NF-κB cytoplasmic retention. Data (*n* = 4) were expressed as the mean (± SEM). *p < 0.05, **p < 0.01 vs control. #*p* < 0.05, ##*p* < 0.01 vs RS-LPS. One-way ANOVA with Tukey’s post hoc test

**Fig. 3:**
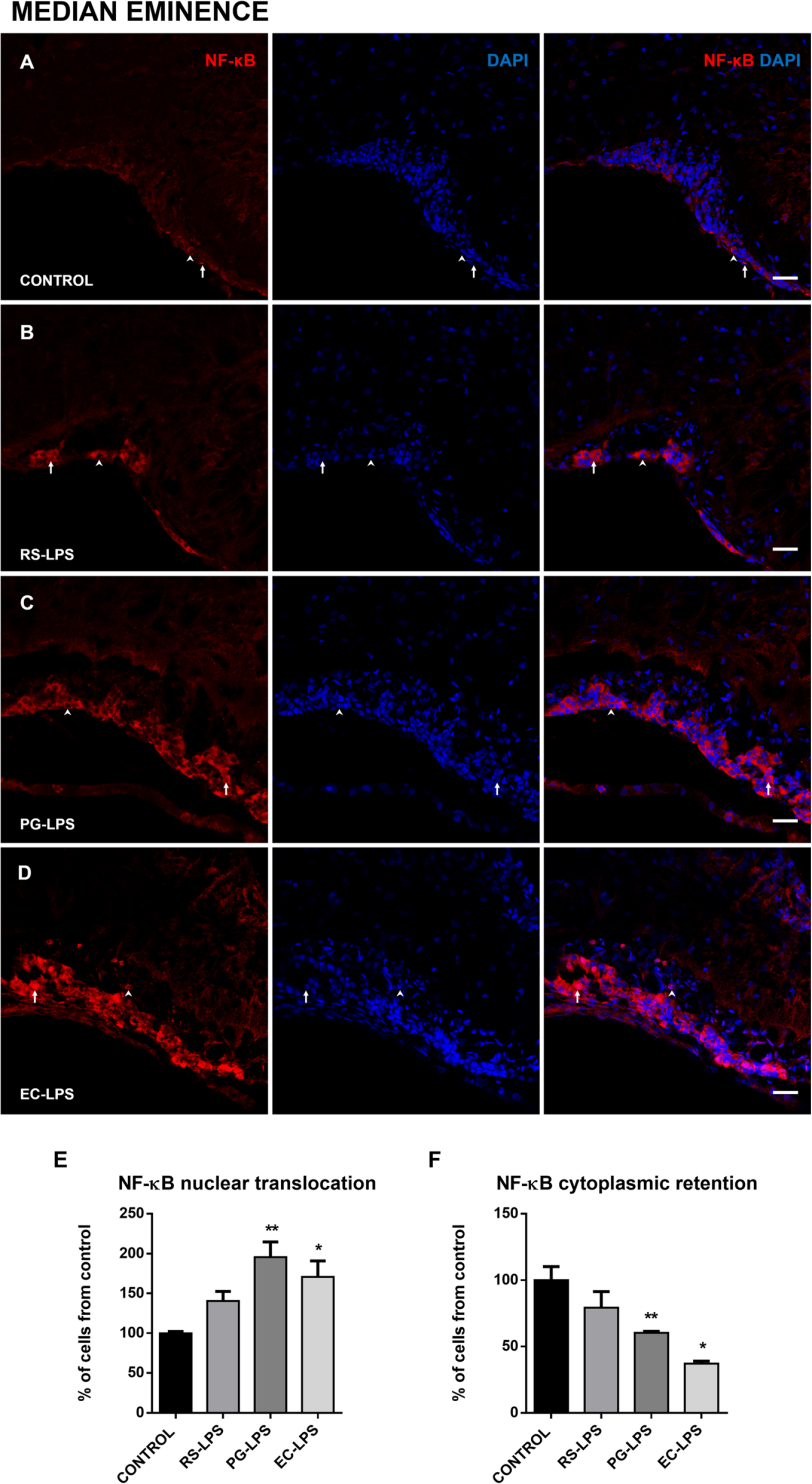
NF-κB cellular localization in the ME of rats i.p. treated with TLR-4 agonists and antagonist LPS. Immunofluorescences of NF-κB in rat ME sections were performed. **a** The control group, **b** RS-LPS i.p. group, **c** PG-LPS i.p. group, and **d** EC-LPS i.p. group. In all cases, red corresponds to NF-κB immunosignal and blue DAPI staining in the nucleus. Arrows indicate red and blue immunosignal overlapping or NF-κB nuclear translocation. Head arrows indicate red immunosignal surrounding blue signal NF-κB cytoplasmic retention. Scale bars = 20 μm. **e** Quantitative analysis of the percentage of NF-κB nuclear translocation. **f** Quantitative analysis of the percentage of NF-κB cytoplasmic retention. Data (*n* = 4) were expressed as the mean (± SEM). *p < 0.05, **p < 0.01, vs control. One-way ANOVA with Tukey’s post hoc test

Similarly, NF-κB nuclear translocation in the EC-LPS group was greater than control (*p* < 0.05) and RS-LPS (*p* < 0.05) groups in the SFO but cytoplasmic retention significantly decreased in PG-LPS (*p* < 0.005) and EC-LPS (*p* < 0.05) compared to control and RS-LPS groups (Fig. 2f). Finally, in the ME, it was observed that NF-κB nuclear translocation increased in EC-LPS (*p* = 0.01) and PG-LPS (*p* = 0.05) (Fig. 3e). Cytoplasmic retention of NF-κB decreased in EC-LPS (*p* = 0.01) and PG-LPS (*p* = 0.05) (Fig. 3f).

AP, SFO, and ME microglial cells change their morphological shape depending on the TLR4 agonist and antagonist lipopolysaccharides administered.

**Fig. 4:**
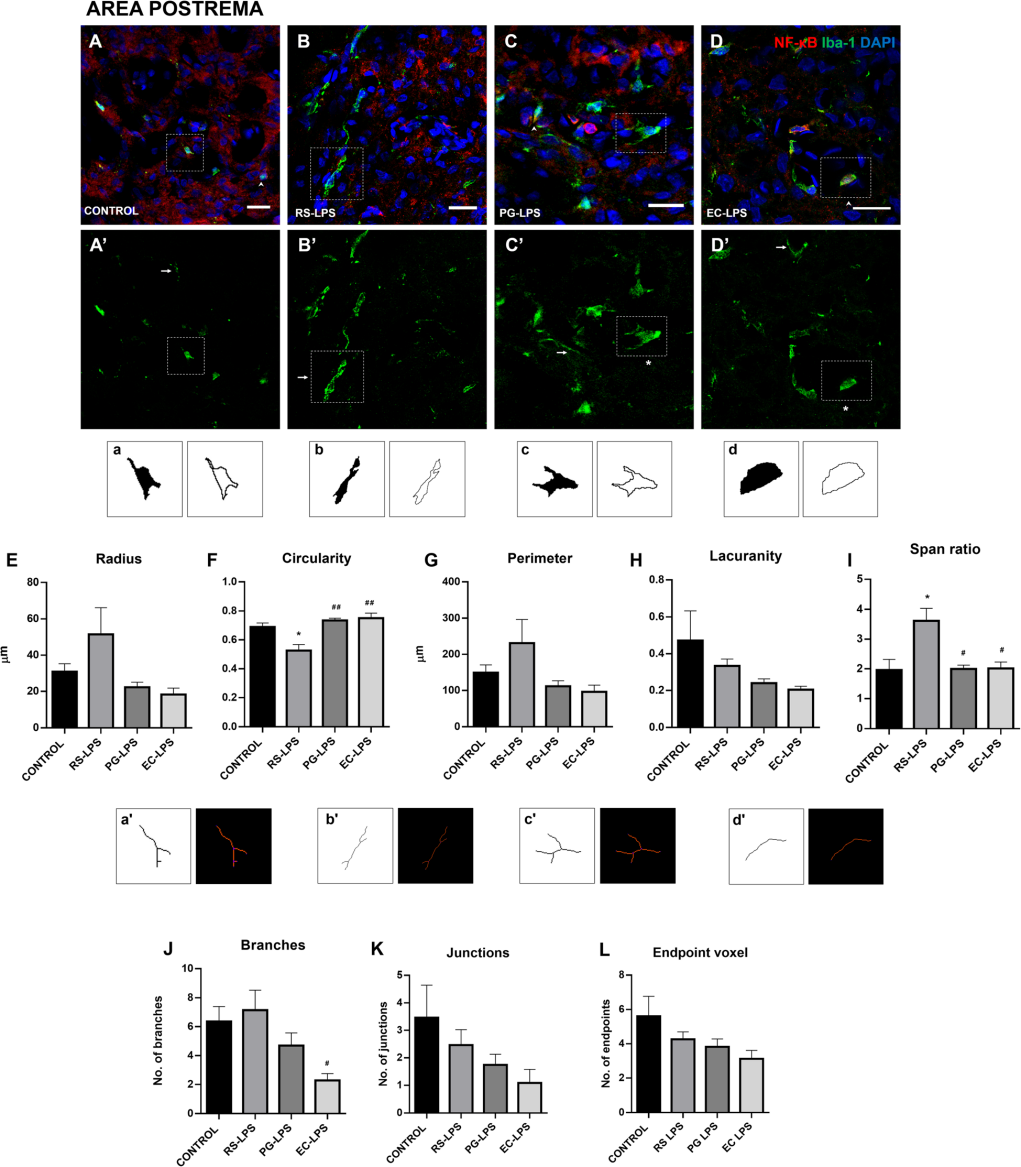
Microglia complexity and ramification in the AP of rats i.p. treated with TLR-4 agonists and antagonist LPS. Immunofluorescence of NF-κB and Iba-1 in rat area postrema sections were performed. **A** The control group, **B** RS-LPS i.p. group, **C** PG-LPS i.p. group, and **D** EC-LPS i.p. group. In all cases, red corresponds to NF-κB immunosignal, green to iba-1 immunosignal and blue DAPI staining in the nucleus. Scale bars = 20 μm. Rectangles in dashed line indicate representative cells in each group. **A’**, **B’**, **C’** and **D’** show only green immune staining. Head arrows indicate NF-κB and Iba-1 positive cells (**C** and **D** panels). Arrows indicate elongated morphology of Iba-1 positive cells (**A’**, **B’**, **C’** and **D’** panels). Asterisks indicate ameboid morphology of Iba-1 positive cells indicate (**C’** and **D’** panels). **a**, **b**, **c**, and **d** binary and outline images of the microglia indicated in the rectangles from images **A’**, **B’**, **C’** and **D’**. Statistical analysis of microglia **E** Maximum radius from the center of the cell, **F** circularity, **G** perimeter, **H** lacunarity, and **I** Span ratio. **a’**, **b’**, **c’**, and **d’** skeletonized binary images and tag skeletonized processes as orange, endpoints as blue, and junctions as purple of the microglia indicated in the rectangles from images **A’**, **B’**, **C’** and **D’**. Statistical analysis of microglia **J** branches **K** junctions, and (**L**) endpoint voxel. Data (*n* = 4) were expressed as the mean (± SEM). *p < 0.05 vs control. #*p* < 0.05 ##*p* < 0.01 vs RS-LPS. One-way ANOVA with Tukey’s post hoc test

**Fig. 5:**
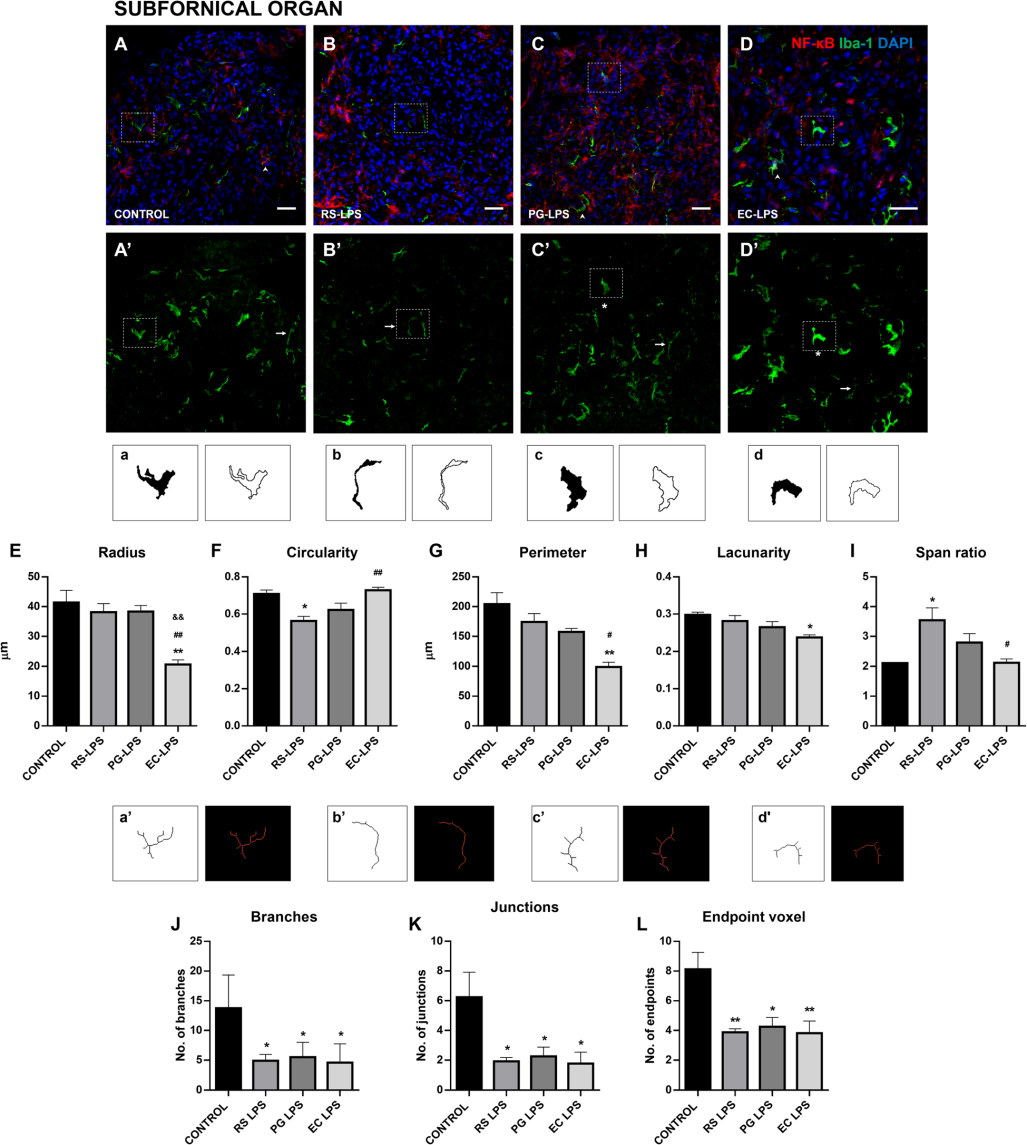
Microglia complexity and ramification in the SFO of rats i.p. treated with TLR-4 agonists and antagonist LPS. Immunofluorescence of NF-κB and Iba-1 in rat SFO sections were performed. **A** The control group, **B** RS-LPS i.p. group, **C** PG-LPS i.p. group, and **D** EC-LPS i.p. group. In all cases, red corresponds to NF-κB immunosignal, green to iba-1 immunosignal and blue DAPI staining in the nucleus. Scale bars = 20 μm. Rectangles in dashed line indicate representative cells in each group. **A’**, **B’**, **C’**, and **D’** show only green immune staining. Head arrows indicate NF-κB and Iba-1 positive cells (**A**, **C**, and **D** panels). Arrows indicate elongated morphology of Iba-1 positive cells (**A’**, **B’**, **C’**, and **D’** panels). Asterisks indicate ameboid morphology of Iba-1 positive cells indicate (**C’** and **D’** panels). **a**, **b**, **c**, and **d** binary and outline images of the microglia indicated in the rectangles from images **A’**, **B’**, **C’**, and **D’**. Statistical analysis of microglia **E** maximum radius from the center of the cell, **F** circularity, **G** perimeter, **H** lacunarity. and **I** span ratio. **a’**, **b’**, **c’**, and **d’** skeletonized binary images and tag skeletonized processes as orange, endpoints as blue, and junctions as purple of the microglia indicated in the rectangles from images **A’**, **B’**, **C’**, and **D’**. Statistical analysis of microglia **J** branches, **K** junctions, and **L** endpoint voxel. Data (*n* = 4) were expressed as the mean (± SEM). *p < 0.05, **p < 0.01, vs control. #*p* < 0.05, ##*p* < 0.01 vs RS-LPS. &&*p* < 0.01 vs PG-LPS. One-way ANOVA with Tukey’s post hoc test

**Fig. 6:**
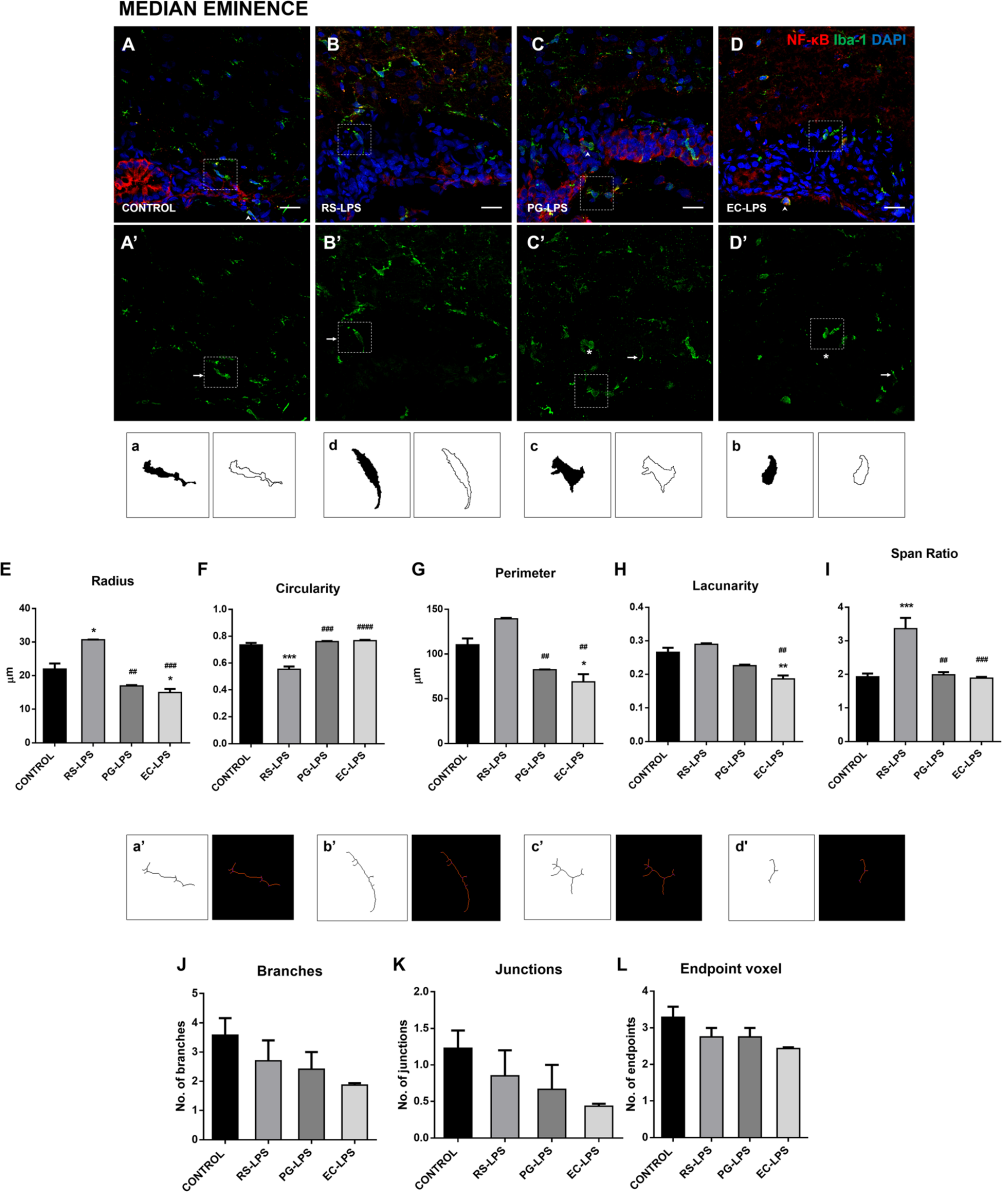
Microglia complexity and ramification in the ME of rats i.p. treated with TLR-4 agonists and antagonist LPS. Immunofluorescence of NF-κB and Iba-1 in rat ME sections were performed. **A** The control group, **B** RS-LPS i.p. group, **C** PG-LPS i.p. group, and **D** EC-LPS i.p. group. In all cases, red corresponds to NF-κB immunosignal, green to iba-1 immunosignal and blue DAPI staining in the nucleus. Scale bars = 20 μm. Rectangles in dashed line indicate representative cells in each group. **A’**, **B’**, **C’**, and **D’** show only green immune staining. Head arrows indicate NF-κB and Iba-1 positive cells (**A**, **C**, and **D** panels). Arrows indicate elongated morphology of Iba-1 positive cells (**A’**, **B’**, **C’**, and **D’** panels). Asterisks indicate ameboid morphology of Iba-1 positive cells indicate (**C’** and **D’** panels). **a**, **b**, **c**, and **d** binary and outline images of the microglia indicated in the rectangles from images **A’**, **B’**, **C’**, and **D’**. Statistical analysis of microglia **E** maximum radius from the center of the cell, **F** circularity, **G** perimeter, **H** lacunarity, and **I** span ratio. **a’**, **b’**, **c’**, and **d’** skeletonized binary images and tag skeletonized processes as orange, endpoints as blue, and junctions as purple of the microglia indicated in the rectangles from images **A’**, **B’**, **C’**, and **D’**. Statistical analysis of microglia **J** branches, **K** junctions, and **L** endpoint voxel. Data (*n* = 4) were expressed as the mean (± SEM). *p < 0.05, **p < 0.01, ***p < 0.001, vs control. ##*p* < 0.01, ###*p* < 0.001 vs RS-LPS. One-way ANOVA with Tukey’s post hoc test

The microglia marker, Iba-1, was used to evaluate whether morphological changes appear in these cells and their inflammatory response was evaluated through NF-κB cellular localization (Fig. 4 A–D, Fig. 5 A–D, and Fig. 6 A–D). The binary and outlined shape were used for fractal analysis in order to evaluate complexity and cell shape. The parameters of radius, circularity, perimeter, lacunarity, and span ratio were useful to measure the distinctive morphology of microglia from the CVOs, not often seen in other well-studied structures such as the frontal cortex.

In the AP, microglia radius registered no significant changes (Fig. 4E). On the other hand, circularity was decreased in the RS-LPS group compared to control (*p* < 0.05), PG-LPS (*p* < 0.001), and EC-LPS groups (*p* < 0.001) (Fig. 4F). In the case of perimeter and lacunarity, no significant changes were observed (Fig. 4G, H). The span ratio, a cell shape or elongation measurement was greater in RS-LPS compared to control (*p* < 0.05), PG-LPS (*p* < 0.001), and EC-LPS groups (*p* < 0.001) (Fig. 4I). The skeleton analysis was used to evaluate changes in the number of branches, junctions, and endpoints and to determine the effect of the different LPS molecules on the typical ramified structure of the microglial cells. The number of branches was lower in EC-LPS compared to the RS-LPS group (*p* < 0.05) (Fig. 4J). In the case of the number of junctions and endpoints, no changes were observed (Fig. 4K, L).

The effects on morphology changes were observed in SFO and ME as well. In the SFO radius, it was observed that the radius decreased in the EC-LPS group compared to control (*p* < 0.01), RS-LPS (*p* < 0.01), and PG-LPS groups (*p* < 0.01) (Fig. 5E). Circularity was lower in RS-LPS group compared to control (*p* < 0.05) and EC-LPS groups (*p* < 0.01) (Fig. 5F). The perimeter was lower in RS-LPS compared to control (*p* < 0.01) and RS-LPS groups (*p* < 0.05) (Fig. 5G). Lacunarity showed no changes (Fig. 5H). In the case of span ratio, the RS-LPS group was significantly greater than control (*p* < 0.05) and EC-LPS groups (*p* < 0.05) (Fig. 5I).

The skeleton analysis showed differences between AP microglial cells. The number of branches was lower in RS-LPS (*p* < 0.05), PG-LPS (*p* < 0.05), and EC-LPS groups (*p* < 0.01) compared to the control group (Fig. 5J). The number of junctions was lower in RS-LPS (*p* < 0.05), PG-LPS (*p* < 0.05), and EC-LPS (*p* < 0.05) compared to the control group (Fig. 5K), and the endpoint was lower in RS-LPS (*p* < 0.01), PG-LPS (*p* < 0.05), and EC-LPS (*p* < 0.01) compared to the control group (Fig. 5L).

In the ME, the radius decreased in the EC-LPS group compared to control (*p* < 0.05), RS-LPS (*p* < 0.01), and PG-LPS groups (*p* < 0.001) (Fig. 6E). Circularity was lower in the RS-LPS group compared to control (*p* < 0.001), PG-LPS (*p* < 0.001), and EC-LPS groups (*p* < 0.0001) (Fig. 6F). The perimeter was lower in EC-LPS compared to control (*p* < 0.05) and RS-LPS groups (*p* < 0.01), and PG-LPS was also lower than the RS-LPS group (*p* < 0.01) (Fig. 6G). Lacunarity was lower in EC-LPS compared to control (*p* < 0.01) and RS-LPS groups (*p* < 0.01) (Fig. 6H). Span ratio was greater in RS-LPS compared to control (*p* < 0.001), PG-LPS (*p* < 0.01), and EC-LPS groups (*p* < 0.001) (Fig. 6I). The skeleton analysis showed no significant changes between groups in the number of branches, junctions nor endpoints (Fig. 6J-L).

Other cellular types of CVOs have no response to acute i.p. LPS administration.

Aiming to identify other cell types besides microglia that could respond to LPSs, perivascular macrophages, neurons, and circulating leukocytes were identified in the CVOs. In the AP and SFO, no NF-κB signal was observed in neurons visualized through NeuN marker (Additional file 1: Figure S1 and Figure S2, respectively). In the area of the fenestrated vessel of the ME, no neurons were found (data not shown). In the case of perivascular macrophages, visualized through the CD163 marker, this cell type was present only in the AP and most of the cells registered positive for the NF-κB signal (Additional file 1: Figure S3), but not in the SFO or in the ME (data not shown). Circulating leukocytes were marked with CD45. This cell type was found only in the AP and ME (Additional file 1: Figure S4 and Figure S5, respectively). There were no differences in the quantity of leukocyte infiltration between groups, or in NF-κB immunosignal, mainly found translocated in the nucleus in all groups.

## Discussion

This study provides evidence about how bacterial LPSs with different endotoxic properties cause distinct effects on CVOs. Most of the experimental (in vivo or in vitro) studies use LPS derived from *E. coli* (the highest endotoxic molecule) as an neuroinflammatory inducer; however, in physiological conditions, CVOs might be exposed to LPSs from different gram-negative species present in gut, mouth, or skin microbiota, each of them with different compositions and structures, and with potential to induce high or low inflammatory responses.

Our results of NF-κB activation in CVOs demonstrate that the immunosignal of this master immune regulator is distributed in the cytoplasm and in the nucleus. Systemic presence of EC-LPS increases nuclear translocation in the three CVOs as several studies have confirmed [37, 38] [4]. However, our study focused on evaluating the effects of an opportunistic bacterial endotoxin such as PG-LPS on the activation of innate immune response mediated by NF-κB in the CVOs. Results showed that in the secretory CVO ME, PG-LPS induces a statistically significant increase in nuclear translocation, different from the AP and the SFO. In oral epithelial membranes*, P. gingivalis* can up-regulate the expression of downstream genes involved in TLR/NF-κB—driven pro-inflammatory response [39]. Our findings are consistent with [40], showing that, in murine models, PG-LPS induces a weak pro-inflammatory response. These authors state that PG-LPS acts exclusively through TLR4 and is recognized in a different way in mouse and human TLR4 both in vitro and in vivo. The predominant effect of PG-LPS on the ME through NF-κB nuclear translocation could be related to the fact that this brain region is a secretory CVO and its vascular permeability for low-molecular-mass molecules is higher compared to sensory CVOs such as the AP and SFO [41].

RS-LPS produced no significant effects on the NF-κB nuclear translocation in the CVOs. Studies have revealed that RS-LPS acts a TLR4 antagonist with analgesic properties in a rat neuropathic model, modulating the release of cytokines [42]. Nonetheless, our results suggest that the existing basal immune activation, evaluated through NF-κB nuclear translocation, in the CVOs was not diminished by the presence of RS-LPS. A recent in vitro study about the effects of RS-LPS in primary mouse microglia demonstrated that this molecule antagonizes EC-LPS when both are co-administrated; however, RS-LPS alone efficiently induces the release of cytokines and chemokines in high doses, which depends on TLR4 [27]. The pretreatment with RS-LPS by intracerebroventricular injection did not significantly alter the LPS-induced increase in the number of NF-κB translocated into the nucleus in microglia of the CVOs [37]. This evidence might corroborate our results according to which systemic administration of RS-LPS has no antagonistic effects in the TLR4 signaling pathway because this could be dependent on a previous inflammatory condition but not on physiological conditions.

An interesting observation was the changes seen in cytoplasmic retention of NF-κB, which occurs in unstimulated cells. NF-κB dimers reside in the cytoplasm bound to their inhibitory subunit, IκB. Upon stimulation, IκBs become phosphorylated, subsequently polyubiquitinated and degraded by the proteasome and NF-κB is translocated to the nucleus [43, 44]. Stimulation with EC-LPS induced a decrease of NF-κB cytoplasmic retention in the three CVOs, and this can be correlated with the increment of NF-κB nuclear translocation. In the case of the PG-LPS group, different effects were observed in the CVOs: in the AP, a significant increase in cytoplasm was found, probably related to an overexpression of the subunit p65 as detected in this study [45]; nonetheless, in the SFO and ME, a decrease in cytoplasmic retention of NF-κB effect was observed, similar to EC-LPS. In the ME, cytoplasmic retention in the group of RS-LPS showed no significant changes.

We have not conducted the usual microglial morphological analysis. Microglia from CVOs are low ramified cells in physiological conditions compared to other brain regions such as the cortex, which made it necessary in this study to analyze parameters based on complexity and shape of the cells using a fractal analysis, such as circularity, radius and perimeter, lacunarity, and span ratio. Circularity determines roundness, increased in amoeboid-like cells. The radius of the cell indicates the maximum radius from the center of the cell, increased in large and elongated cells. The perimeter of the cell can increase or decrease depending on cell shape, whether this is elongated or amoeboid, respectively. Lacunarity refers to the degree of inhomogeneity and translational and rotational invariance in an image, where low lacunarity implies homogeneity and where rotating the image will not change it significantly [45]. Span ratio describes the shape of the outline of the cell where a high span ratio indicates elongation of the cell [46].

EC-LPS group showed significant changes in microglia morphology in the SFO and ME; however, in the AP, the same results tended to be obtained. A lower radius from the center and perimeter indicates that microglial cells exposed to this molecule were smaller than those from the control group. A decrease in lacunarity shows that cells had a homogeneous outline. These significant changes indicate that microglia in the CVOs exposed to EC-LPS tend to have an ameboid shape due to their activation. In other studies, similar results have been obtained after exposure to EC-LPS in the AP where microglia are activated [28] or showed less ramification compared to the adjacent structure, the nucleus of the solitary tract [47]. Administration of PG-LPS did not significantly alter the morphology of microglial cells; however, a tendency was observed in the reduction of radius from the center, perimeter and lacunarity parameters and in all the CVOs tested, as well as in the group of rats injected with EC-LPS. These results indicate that microglia in the CVOs after administration of PG-LPS tend to change their morphology to an ameboid shape. As described by [40], PG-LPS is exclusively recognized by TLR4, inducing low immune activation in mice, and this could be related to low TLR4 and MD2 affinity to Penta-acylated *P. gingivalis* lipid A, producing differences in expression levels of pro-inflammatory molecules. A recent study in mice, injecting 5 mg/kg (i.p.) of PG-LPS, a higher dose than the one used in our study, reported that microglia in the hippocampus and cortex was activated, and impaired spatial learning and memory in behavioral tests [20]. In addition, it has been described that PG-LPS stimulation is more effective, in terms of cytokine release, in middle-aged mice than young counterparts, which suggests a possible relationship to human adults with periodontal inflammation that can be uncontrollable [48]. Probably, to observe activation of the inflammatory response in the brain caused by PG-LPS, higher doses of this molecule in aged animals would be necessary.

The most unexpected results were the observation of a radical microglial morphological change in the RS-LPS group, an increase of cell radius from the center, loss of circularity, and a significant increase of span ratio, indicating elongation of the cells in all tested CVOs. This type of morphology has been described as “rod” microglia and is associated with brain infections, traumatic brain injury and neurodegenerative disorders [49]. Franz Nissl (1860-1919) and Alois Alzheimer (1864-1915) were the first to describe the presence of rod cells in pathological tissues, but Pío del Río-Hortega (1882-1945) was the first to show that these pathological cells originated from microglia [50]. Nowadays, the function and activity of “rod” microglia are still unknown, although in traumatic brain injury, neuronal damage causes the formation of “rod” microglia promoting astrogliosis and persistent neuroinflammation [51]. In addition, a study carried out in a series of human autopsies showed that older chronological age was a strong predictor for the presence of rod-shaped microglia, even during the control of Alzheimer’s disease [52]. Indeed, microglia from CVOs in normal conditions are continuously activated without any pathological stimulation, exhibiting phagocytic and antigen-presenting activities, and even amoeboid form [53]. Thus, the peripheral presence of RS-LPS induces rod-microglia morphology, suggesting a possible noxious effect in microglia function or even in neurons affecting the immune response to immunological and damaging insults.

An interesting finding was to observe that not all microglial cells expressed and translocated the NF-κB p65 subunit, even in those activated microglia with amoeboid like-shape in the EC-LPS and PG-LPS groups. It is clear that the three LPS treatments induced effects on microglial morphology; however, LPS signaling over in these cells not necessarily could be related to the canonical NF-κB pathway where p50 and p65 nuclear translocation is produced [54]. It has been described different ways on how LPS can activate an immune response in myeloid and non-myeloid cells, including microglia. For example, the non-canonical NF-κB pathway where p52 and RelB are translocated to the nucleus [55]. In addition, there are other receptors that bind LPS besides the canonical TLR-4. Caspases 4/5/11 are cytosolic LPS receptors, activating through oligomerization with LPS [56]. The receptor for advanced glycation end products (RAGE) can interact with LPS, and this receptor can be responsible for microglial activation and production of proinflammatory mediators in Alzheimer’s disease [57]. This evidence could explain the other type of LPS interaction with microglia without the activation of the canonical NF-κB activation pathway, and the possible LPS binding to other receptors which could be able also to recognize different structures and composition of this bacterial molecule.

There is evidence of harmful microbiota-derived TLR4 antagonistic LPS effects on health promoting the development of autoimmune diseases. In a recent study, it has been found that LPS from *Bacteroides dorei* with TLR4 antagonist properties was abundant in children from countries with an early onset of autoimmune disease. Conversely, LPS from *E. coli* was more prevalent, and prevented the development of the autoimmune disease. The main conclusion of this study was that agonistic LPS with endotoxic properties can prevent autoimmune disease by immuno-stimulation and so-called “endotoxin tolerance” is a factor that contributes to a better “immune education”. On the contrary, antagonistic LPS inhibits immune stimulation and the inflammatory response from other agonist LPS molecules [58].

EC-LPS i.p. or i.c.v. administration is among the most common models of neuroinflammation induction [59]. However, few studies have referred to the effects of a limited variety of LPS molecules and their endotoxic properties in the brain immune response. Our research group has recently shown the infiltration of LPS in the brain possibly transported by plasma lipoproteins, in physiological conditions, in blood-brain-interfaces such as CVOs [5], and recently, the presence of uncovered bacteria in healthy individuals has been reported [48]. These evidences suggest a necessary re-evaluation of the theory of a sterile brain and the immunological privilege of this organ. A microorganism from microbiota or from infections can act directly on the brain through the most abundant molecule in gram-negative bacteria, LPS, modulating the inflammatory response by shaping glial activity. Possibly, the CNS is not excluded from “immune education” by composition microbiota as occurs in the periphery, more specifically, a variation of LPS TLR4 agonistic and antagonistic functions could be responsible for brain immune defense in health and disease.

Further studies about the role of TLR4 agonist and antagonist LPS in the CNS immunity will require to evaluate other types of LPS from different human microbiota gram-negative bacteria involved in healthy and pathological conditions to elucidate their mechanism in the inflammatory response.

In addition, it will be necessary to evaluate the effect of the LPS molecules used in this study on different time stages to assess the response of different inflammatory pathways besides the canonical NF-κB pathway.

It will be also needed a chronic administration to analyze these molecules tolerance response in brain immunity, as well as, to analyze their effect on parenchymal structures, such as the brain cortex and hippocampus.

## Conclusions

In conclusion, our results, as well as other studies, demonstrated that the canonical TLR4 agonist, EC-LPS, activates the inflammatory response in the CVOs through NF-κB and microglia activation, changing their morphology to an ameboid shape. On the other hand, PG-LPS, as a mild TLR4 agonist, only induced NF-κB nuclear translocation in the ME, probably because this secretory CVO has a higher vascular permeability compared to sensory CVOs. There was only a tendency to change microglial morphology into an ameboid shape by PG-LPS administration, maybe because concentration and immune deficiency by aging are involved to induce an inflammatory response in the brain. On the contrary, RS-LPS did not change NF-κB activation in the CVOs. Interestingly, this TLR4 antagonist induced a rod microglia shape, a type of morphology found in severe pathological situations such as traumatic brain injury, infections, or neurodegenerative diseases. Microbiota variation leading to an imbalance, where TLR4 antagonist LPS molecules are increased compared to TLR4 agonist ones, could lead to the development of neurological conditions where rod microglia are involved. The therapeutic use of TLR4 antagonist from LPS or synthetic molecules to treat neuroinflammatory conditions should be carefully revised since the inflammatory response is a strategy of the immune system to attack immunological and damaging insults, microglia activation and NF-κB nuclear translocation in the CVOs are normally present, perhaps because these structures play the role of the first line of immune defense in the brain.

## Supplementary information

**Additional file 1:** Supplementary information.

## Data Availability

The datasets used and/or analyzed during the current study are available from the corresponding author on reasonable request.
