# Supplementary material for: Toll-like receptor 4 agonist and antagonist lipopolysaccharides modify innate immune response in rat brain circumventricular organs
Source: J Neuroinflammation. 2020 Jan 6;17:6. doi: 10.1186/s12974-019-1690-2 (PMC6945636; doi:10.1186/s12974-019-1690-2)
Supplement: Supplementary file 1 — Additional file 1: Supplementary information. [file 12974_2019_1690_MOESM1_ESM.docx]

**tlr4 agonist and antagonist lipopolysaccharides modify innate immuNe response in rat BRAIN circumventricular organs**

Vargas-Caraveo A^1,2^*, Sayd A ^1^, Robledo J^1^, Caso JR^1^, Madrigal JLM^1^, García Bueno B^1^* and Leza JC^1^

^1^ Departamento de Farmacología y Toxicología, Facultad de Medicina, Universidad Complutense de Madrid (UCM); Centro de Investigación Biomédica en Red de Salud Mental (CIBERSAM); Instituto de Investigación Sanitaria Hospital 12 de Octubre (Imas12); Instituto Universitario de Investigación en Neuroquímica UCM; Avda. Complutense s/n, Madrid-28040 (Spain).

^2^ Biological and Health Sciences Division, Metropolitan Autonomous University (UAM), Campus Lerma, 52005 Lerma, Mexico.

* Corresponding authors:

Dr. Alejandra Vargas-Caraveo

e-mail: a_vargas@correo.ler.uam.mx

Dr. Borja García-Bueno

e-mail: [bgbueno@med.ucm.es](mailto:bgbueno@med.ucm.es)

**SUPPLEMENTARY INFORMATION**


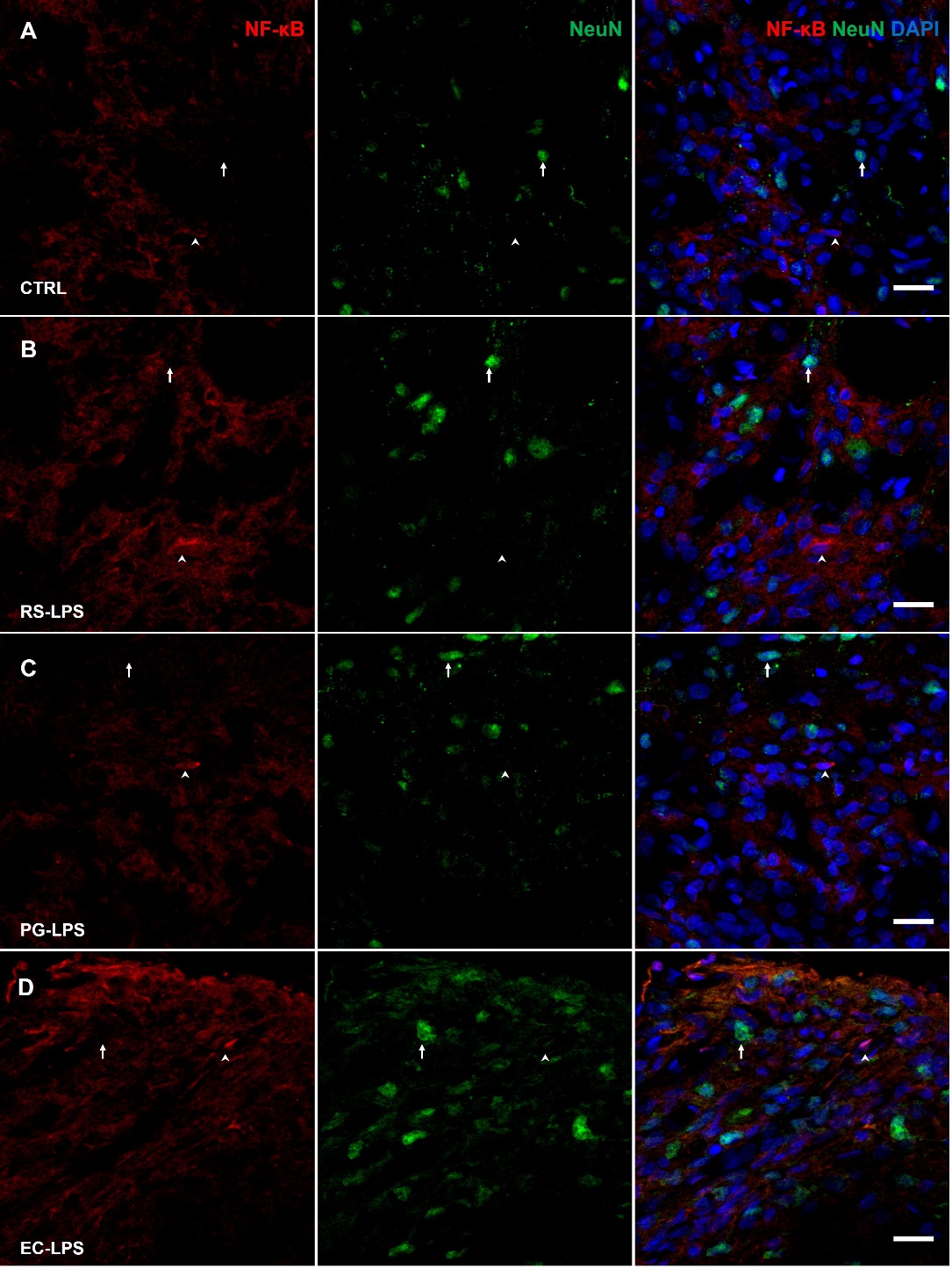


**Figure S1. NF-κB localization in neurons of the AP of rats i.p. treated with** **TLR4 agonists and antagonist LPS.** Immunofluorescences of NF-κB and neuronal marker NeuN in rat AP sections were performed. (A) control group, (B) RS-LPS i.p. group, (C) PG-LPS i.p. group and (D) EC-LPS i.p. group. In all cases, red corresponds to NF-κB immunosignal, green corresponds to soma neurons marked with NeuN and blue DAPI staining in the nucleus. Arrows indicate green immunosignal of NeuN. Head arrows indicate red immunosignal of NF-κB. Scale bars = 20 μm.


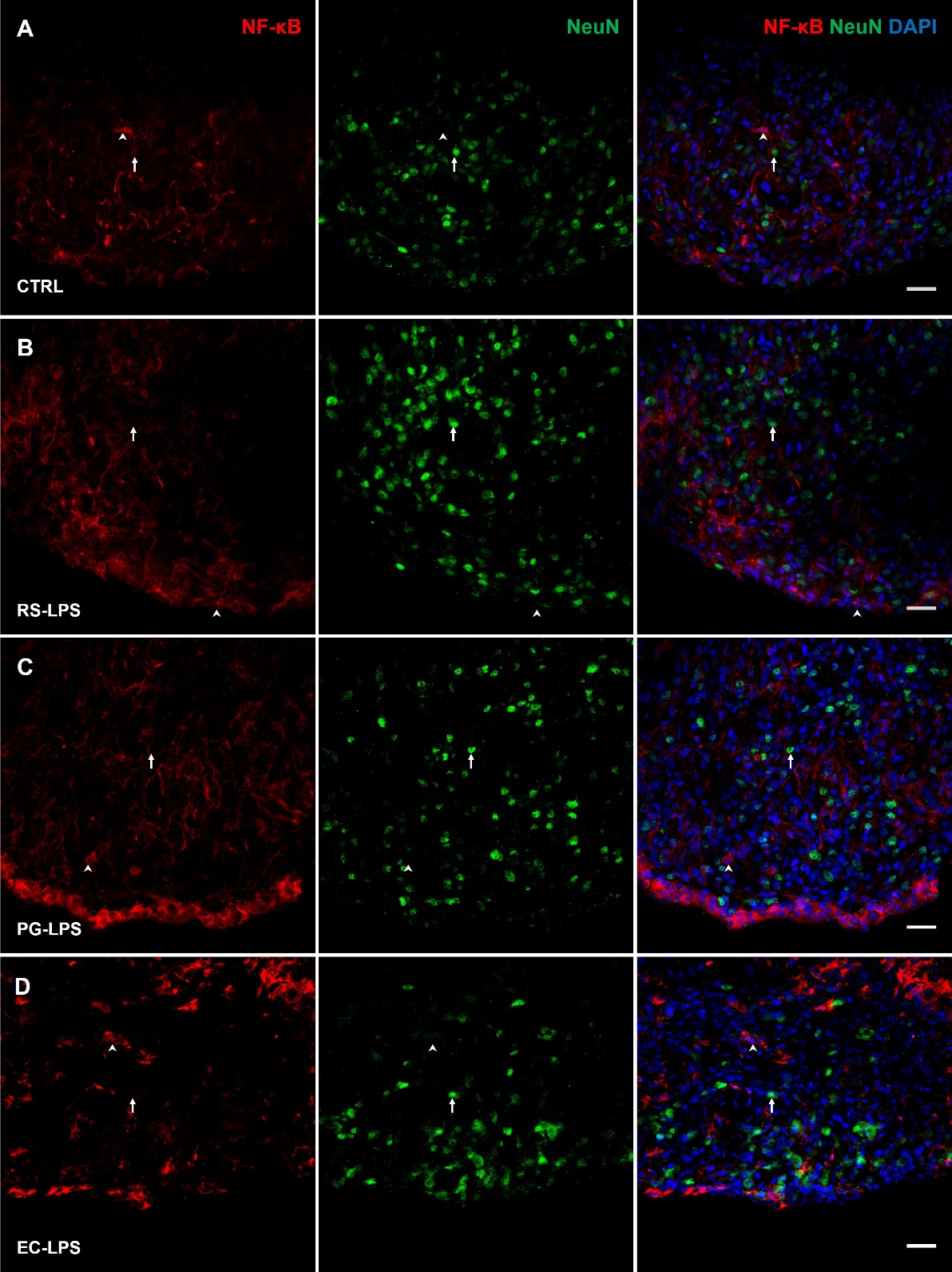


**Figure S2. NF-κB localization in neurons of the SFO of rats i.p. treated with TLR4 agonists and antagonist LPS.** Immunofluorescences of NF-κB and neuronal marker NeuN in rat AP sections were performed. (A) control group, (B) RS-LPS i.p. group, (C) PG-LPS i.p. group and (D) EC-LPS i.p. group. In all cases, red corresponds to NF-κB immunosignal, green corresponds to soma neurons marked with NeuN and blue DAPI staining in the nucleus. Arrows indicate green immunosignal of NeuN. Head arrows indicate red immunosignal of NF-κB. Scale bars = 20 μm.


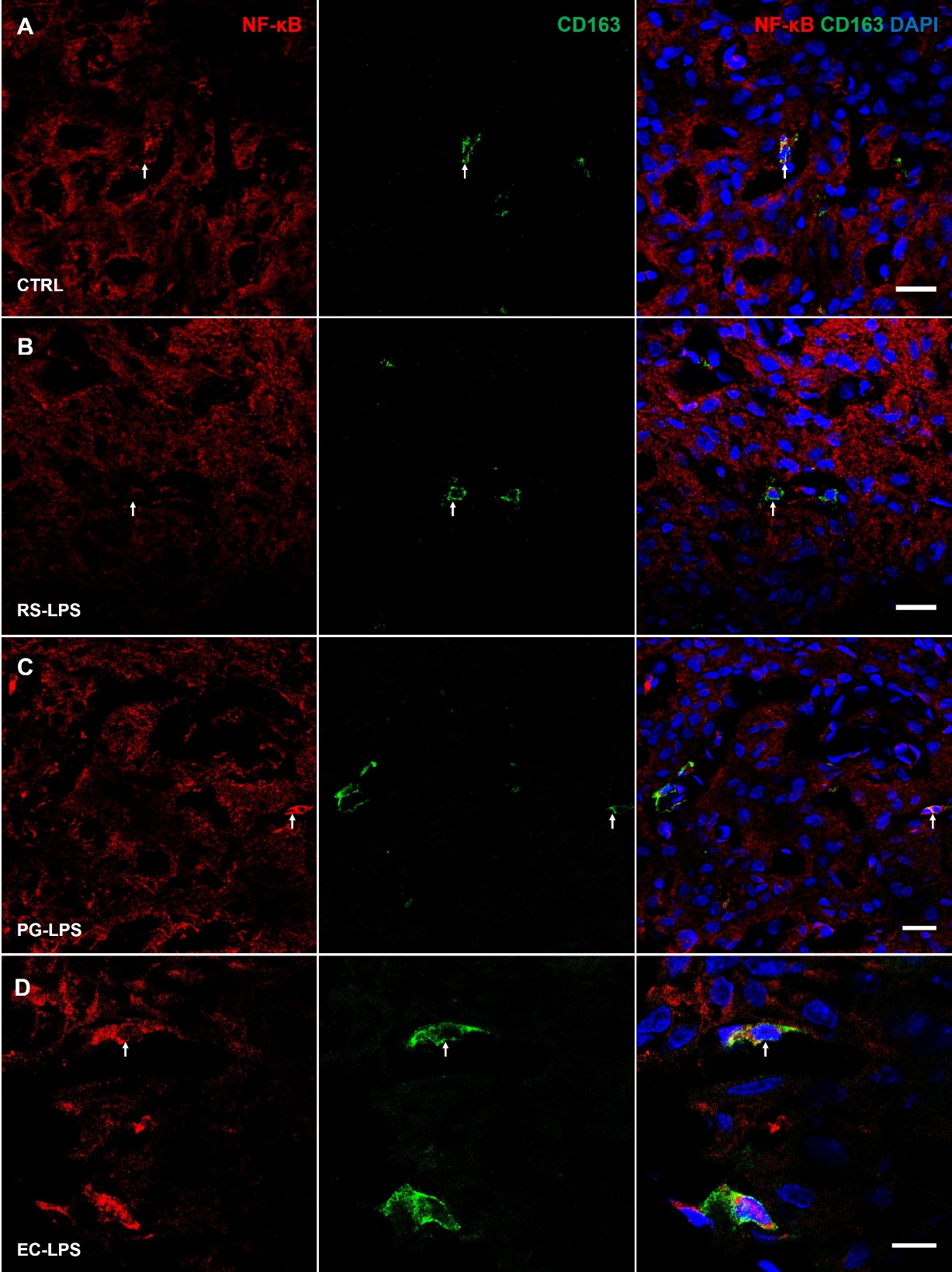


**Figure S3. NF-κB localization in perivascular macrophages of the AP of rats i.p. treated with TLR4 agonists and antagonist LPS.** Immunofluorescences of NF-κB and macrophage marker CD163 in rat AP sections were performed. (A) control group, (B) RS-LPS i.p. group, (C) PG-LPS i.p. group and (D) EC-LPS i.p. group. In all cases, red corresponds to NF-κB immunosignal, green corresponds to CD163 and blue DAPI staining in the nucleus. Arrows indicate positive cells to CD163 with NF-κB immunosignal. Scale bars = 20 μm.


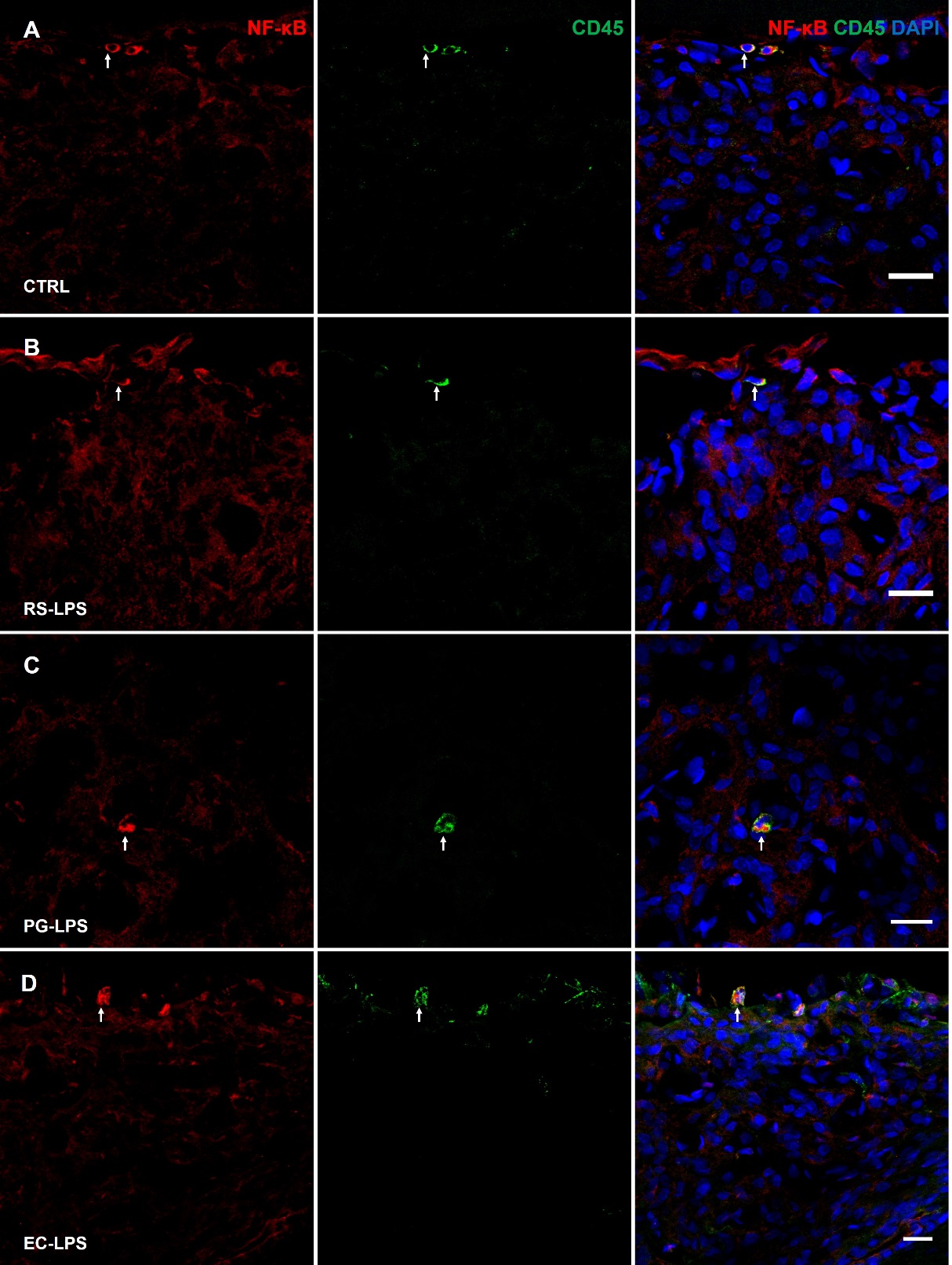


**Figure S4. NF-κB localization in infiltrated leukocytes of the AP of rats i.p. treated with TLR4 agonists and antagonist LPS.** Immunofluorescences of NF-κB and the common leukocyte marker CD45 in rat AP sections were performed. (A) control group, (B) RS-LPS i.p. group, (C) PG-LPS i.p. group and (D) EC-LPS i.p. group. In all cases, red corresponds to NF-κB immunosignal, green corresponds to CD45 and blue DAPI staining in the nucleus. Arrows indicate positive cells to CD45 with NF-κB immunosignal. Scale bars = 20 μm.


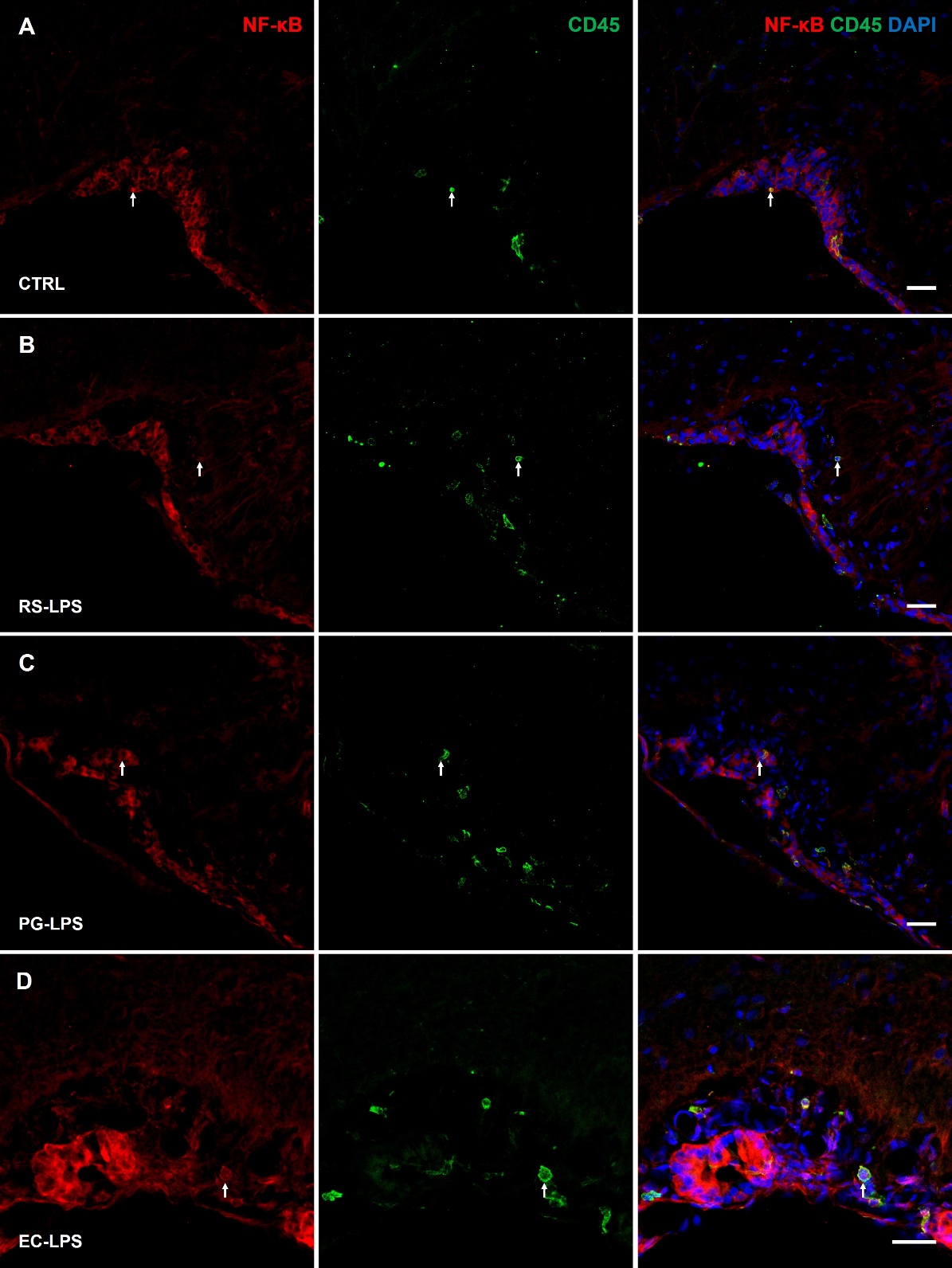


**Figure S5. NF-κB localization in infiltrated leukocytes of the ME of rats i.p. treated with TLR4 agonists and antagonist LPS.** Immunofluorescences of NF-κB and the common leukocyte marker CD45 in rat AP sections were performed. (A) control group, (B) RS-LPS i.p. group, (C) PG-LPS i.p. group and (D) EC-LPS i.p. group. In all cases, red corresponds to NF-κB immunosignal, green corresponds to CD45 and blue DAPI staining in the nucleus. Arrows indicate positive cells to CD45 with NF-κB immunosignal. Scale bars = 20 μm.
